# Central Role of the Actomyosin Ring in Coordinating Cytokinesis Steps in Budding Yeast

**DOI:** 10.3390/jof10090662

**Published:** 2024-09-21

**Authors:** Magdalena Foltman, Alberto Sanchez-Diaz

**Affiliations:** 1 Mechanisms and Regulation of Cell Division Research Unit, Institute of Biomedicine and Biotechnology of Cantabria (IBBTEC), University of Cantabria-CSIC, 39011 Santander, Spain; magdalena.foltman@unican.es; 2 Molecular Biology Department, Faculty of Medicine, University of Cantabria, 39005 Santander, Spain

**Keywords:** cell division, cytokinesis, septin, actomyosin ring, ingression, ingression progression complexes (IPCs), extracellular matrix remodeling, septum formation, budding yeast

## Abstract

Eukaryotic cells must accurately transfer their genetic material and cellular components to their daughter cells. Initially, cells duplicate their chromosomes and subsequently segregate them toward the poles. The actomyosin ring, a crucial molecular machinery normally located in the middle of the cells and underneath the plasma membrane, then physically divides the cytoplasm and all components into two daughter cells, each ready to start a new cell cycle. This process, known as cytokinesis, is conserved throughout evolution. Defects in cytokinesis can lead to the generation of genetically unstable tetraploid cells, potentially initiating uncontrolled proliferation and cancer. This review focuses on the molecular mechanisms by which budding yeast cells build the actomyosin ring and the preceding steps involved in forming a scaffolding structure that supports the challenging structural changes throughout cytokinesis. Additionally, we describe how cells coordinate actomyosin ring contraction, plasma membrane ingression, and extracellular matrix deposition to successfully complete cytokinesis. Furthermore, the review discusses the regulatory roles of Cyclin-Dependent Kinase (Cdk1) and the Mitotic Exit Network (MEN) in ensuring the precise timing and execution of cytokinesis. Understanding these processes in yeast provides insights into the fundamental aspects of cell division and its implications for human health.

## 1. Introduction

**Figure 1:**
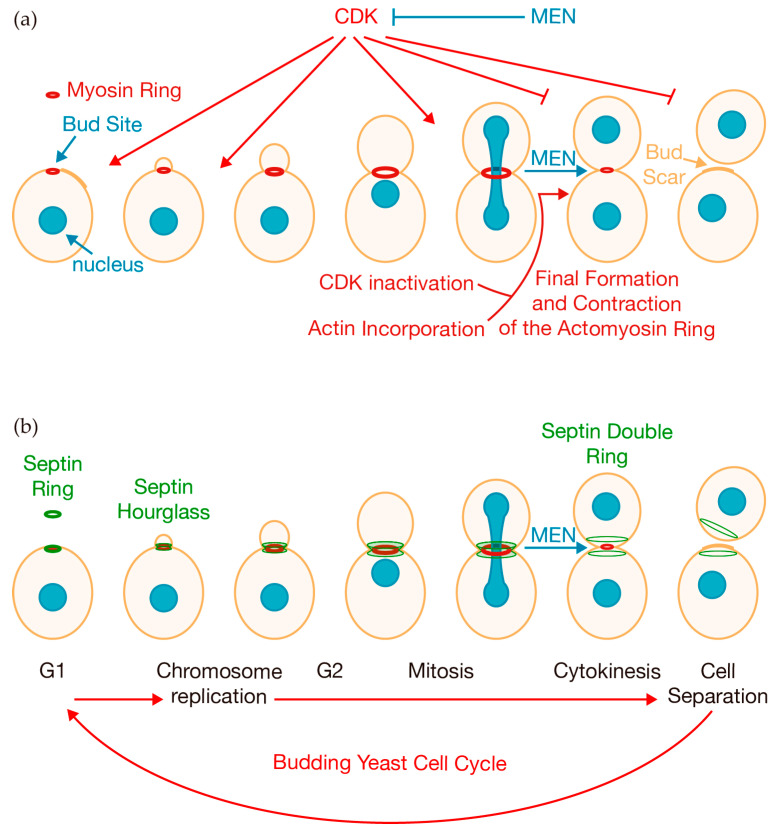
Illustration of the different phases of the budding yeast cell cycle. (**a**) Cells assemble an actomyosin ring early in the cell cycle at the site of the presumptive bud site. Kinase activity associated with CDK and the signaling cascade known as MEN ensure that cytokinesis occurs once cells have segregated their duplicated chromosomes. CDK activity needs to be downregulated to allow for actin incorporation and the completion of contractile ring formation, which is then followed by the contraction of the actomyosin ring. (**b**) Cells also build a scaffolding structure, known as the septin ring, at the presumptive bud site, which undergoes structural changes throughout the cell cycle. This structure transforms into an hourglass shape and, just before the onset of cytokinesis, the septin hourglass splits into two distinct rings.

Eukaryotic cells undergo a highly regulated process known as cytokinesis at the end of mitosis to ensure the proper distribution of the duplicated genome and organelles to the two daughter cells (Figure 1). When cell division fails, it can result in the creation of genetically unstable tetraploid cells, which have the potential to initiate uncontrolled cell proliferation and tumorigenesis [1,2,3]. The molecular mechanisms of cytokinesis are highly conserved from yeast to human cells and involve the coordination of multiple cellular structures and signaling pathways [4,5,6]. The budding yeast *Saccharomyces cerevisiae* has been extensively used as a model organism to study various molecular mechanisms, including the cell cycle. Cytokinesis has been particularly well studied in budding yeast, providing a comprehensive understanding of the fundamental mechanisms that govern cytokinesis in eukaryotic cells.

Key to cytokinesis is the actomyosin ring (Figure 1a), a dynamic structure that is assembled and contracts and contains a large number of proteins, including myosin type II and actin filaments [4,5]. Deciding where to place the actomyosin ring has changed more significantly than other aspects of cytokinesis, leading animal cells, fission yeast, and budding yeast to adopt different strategies to complete this process. In animal cells, the mitotic spindle serves as the main source of positional information for cytokinesis. In contrast, fission yeast relies on signals from both the cell poles and the nucleus to accurately place the actomyosin ring [6]. *S. cerevisiae* grows by budding, and this is precisely the point at which new tiny bud emerges and cells build the whole cytokinetic machinery to drive the final physical division (Figure 1). The new bud emerges next to the mark left by the previous cell division and separation in the mother cell, referred to as the bud scar (Figure 1a).

The assembly of this sophisticated apparatus begins with the formation of a septin scaffold, which serves as a platform for the recruitment of other cytokinetic components [7]. Early in the cell cycle, septins are recruited to the presumptive bud site, where they assemble into an initial septin ring (Figure 1b). After bud formation, the septin ring transitions into an hourglass-shaped structure (Figure 1b). As cytokinesis begins, the septin hourglass undergoes major structural changes, splitting into two separate rings (Figure 1b). A step-wise process allows cells to build a ring ready to contract at the end of mitosis (Figure 1).

The efficient execution of cytokinesis requires the accurate coordination between the contraction of the actomyosin ring, the ingression of the plasma membrane, and the remodeling of the extracellular matrix [4,5,6,8]. The plasma membrane forms a physical barrier between the two daughter cells. This plasma membrane is delivered as part of vesicles through the secretory pathway. Additionally, these vesicles transport essential cargoes that must be integrated into the molecular machinery driving cytokinesis [5,9]. Yeast, being unicellular organisms, are surrounded by a rigid extracellular matrix known as the cell wall, which provides the necessary structural support and protection for their survival. During cytokinesis, yeast cells synthesize a special layer of extracellular matrix between the two daughter cells known as the primary septum, which is essential for cytokinesis and is made of chitin [4,5]. Additionally, yeast cells form secondary septa at both sides of the primary septum [10] The primary septum is eventually digested to allow the separation of the two daughter cells [10,11], a process that is coordinated with cellular growth [12,13].

Timing of cytokinesis is essential to ensure cell division occurs only when cells have segregated their duplicated chromosomes (Figure 1a). The kinase activity of Cyclin-Dependent Kinases (CDKs) is essential for driving cell cycle progression [14,15,16,17]. In budding yeast, the primary CDK is Cdk1, also known as Cdc28. This catalytic subunit forms complexes with various cyclins at different stages of the cell cycle, phosphorylating key substrates to facilitate chromosome replication and segregation (Figure 1). Interestingly, the same kinase activity that promotes cell cycle progression can also inhibit late cell cycle events such as exit from mitosis, cytokinesis, and cell separation [11,18,19,20,21] (Figure 1a). To complete each round of cell division, cells must downregulate CDK activity to lift this inhibition (Figure 1a). A second signaling cascade, known as the Mitotic Exit Network (MEN), is triggered when cells segregate their chromosomes and control the timing of mitotic exit and cytokinesis (Figure 1a). This cascade includes the small GTPase Tem1 and protein kinases such as Cdc15, Dbf2, and Dbf20, along with a few regulatory proteins [22]. MEN activity ultimately releases the evolutionarily conserved phosphatase Cdc14 from the nucleolus, allowing it to dephosphorylate substrates that promote mitotic exit and facilitate efficient and rapid cytokinesis, thereby initiating a new cell cycle [21,22,23,24,25]. As described below, Cdc14 dephosphorylates key cytokinetic proteins. Furthermore, MEN kinases play a key role in driving cytokinesis [26,27] (Figure 1).

This review explores the molecular mechanisms underlying the construction and function of the actomyosin ring in budding yeast. It examines the steps leading to the formation of the septin scaffold, the recruitment and regulation of actomyosin ring components, and the coordination of ring contraction with plasma membrane ingression and extracellular matrix remodeling.

## 2. Positioning the Contractile Ring in the Cell: The Septin Scaffold

Some of the first components to assemble into the ring are the septins, a conserved family of GTP-binding proteins essential for various cellular processes, particularly cytokinesis [7]. They were first identified by Professor Hartwell, who described numerous genes involved in cell cycle regulation [28,29,30]. Mitotic septins include the proteins Cdc3, Cdc10, Cdc11, and Cdc12, originally described in Hartwell’s screen, and Shs1, isolated a few decades later [31,32]. Septins are fully conserved from yeast to humans [33,34], and alterations in septin function are associated with human pathologies such as cancer, cardiovascular, reproductive, and neurodegenerative diseases [35].

**Figure 2:**
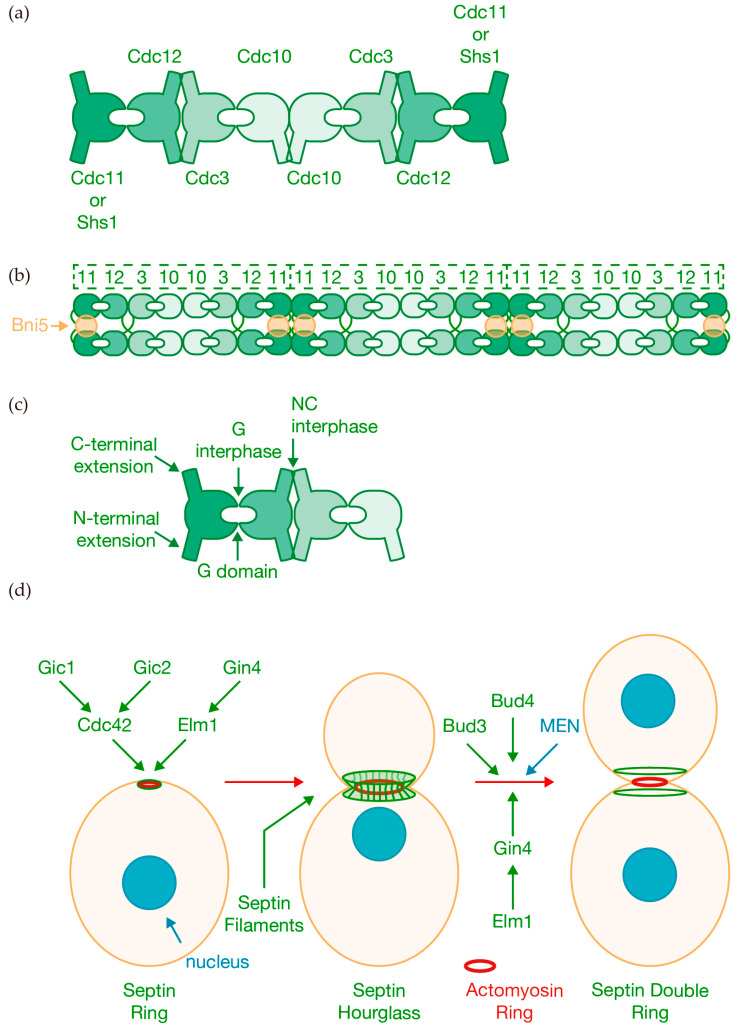
Septin filaments provide structural support to cytokinesis. (**a**) The hetero-octameric rod is formed by the interaction of individual septins in the order Cdc11–Cdc12–Cdc3–Cdc10–Cdc10–Cdc3–Cdc12–Cdc11. The septin found at both ends can be either Cdc11 or Shs1. (**b**) Septin filaments associate through lateral interactions involving the C-terminal extensions of Cdc3 and Cdc12, forming long parallel filaments. This process is regulated by the septin-binding protein Bni5, which interacts with the C-terminal extensions of Cdc11 and Shs1 to control filament pairing. (**c**) Illustration of the interactions within the basic septin heteromeric rods. (**d**) Representation of major septin architectural transformations during the cell cycle and the key proteins that drive its assembly and remodeling.

In budding yeast, septins are required for the assembly of the actomyosin ring [36]. The five septins assemble into higher-order oligomers and filamentous polymers, interacting with membranes and the cytoskeleton. Originally, septins were described as neck filaments encircling the structure that connects mother and daughter cells, named the bud neck [37] (Figure 1b). Four septins form a linear, apolar hetero-octameric rod with the order Cdc11–Cdc12–Cdc3–Cdc10–Cdc10–Cdc3–Cdc12–Cdc11, and Cdc11 located at both ends [38,39] (Figure 2a). These rods undergo end-to-end assembly to form filaments (Figure 2b), which associate laterally with other filaments through the interaction of the C-terminal extensions of Cdc3 and Cdc12, resulting in long parallel tracks [38,40] (Figure 2b). Unlike the others, septin Shs1 is non-essential and may replace Cdc11 at the end of the rod, forming octameric rods (Shs1–Cdc12–Cdc3–Cdc10–Cdc10–Cdc3–Cdc12–Shs1) (Figure 2a) that fail to polymerize into filaments, instead engaging in lateral associations leading to the formation of curved bundles [39,40].

Most of our current knowledge of septin molecular structure comes from studying human septins [30,41], though we have molecular details for yeast Cdc11 and the Cdc3-Cdc10 heterodimer [42,43]. The basic heteromeric rods are assembled through the conserved interaction between adjacent GTP-binding domain (G domain) and the amino- and carboxy-terminal extensions (Figure 2c). The polymerization of the monomers requires the formation of two distinct types of interfaces with their adjacent counterparts on either side, designated as NC and G, which alternate sequentially along the filament [30,41,43] (Figure 2c). Using bimolecular fluorescence complementation (BiFC), Weems and McMurray described the sequential pathway for the assembly of septin hetero-octamers in budding yeast [44]. The hydrolysis of GTP by the monomeric Cdc10 drives the formation of the core Cdc10 homodimer. The N-terminal extension of Cdc3 blocks the association between Cdc3 and Cdc10 homodimers until the preceding interaction between Cdc3 and Cdc12 occurs. The slower GTP hydrolysis by monomeric Cdc12, coupled with the specific affinity of Cdc11 for the transient GTP-bound Cdc12, facilitates the assembly of distinct trimeric structures, either Cdc11–Cdc12–Cdc3 or Shs1–Cdc12–Cdc3 [44]. A reduction in the cytosolic GTP/GDP ratio leads to an increased incorporation of Shs1 compared to Cdc11, altering the curvature of the filamentous septin rings [44].

Septin rods form short filaments on the plasma membrane. These filaments can organize into more complex hierarchical structures, including rings, hourglass formations, and gauze-like arrays depending on the cell cycle [45,46]. The plasma membrane functions as a platform, with septins binding membranes containing phosphatidylinositol-4,5-bisphosphate (PIP2) and recognizing membrane curvature, facilitating the organization into higher-order structures that vary along the cell cycle [47,48,49,50] (Figure 2d).

At the beginning of the cell cycle, septins localize at the presumptive bud site, forming an incipient septin ring (Figure 2d). The nascent ring is detected just prior to bud emergence, marking the area where the bud will initially grow and develop into a daughter cell. Components of this nascent ring are highly mobile [51,52]. This process is driven by the small GTP Cdc42 and its effectors, as well as GTPase interacting components (GICs), Gic1 and Gic2 [51,53,54,55,56].

Following the formation of the bud, the septin ring adopts an hourglass configuration (Figure 2d). This collar-shaped structure persists at the bud neck until anaphase, serving as a crucial and highly stable scaffold [51,52]. It supports the assembly of the actomyosin ring, which drives cytokinesis at the end of the cell cycle [7,45,46]. The septin hourglass serves as a membrane-diffusible barrier between the mother and the growing bud [57,58]. Studies have shown that the septin hourglass seems to consist primarily of highly packed paired filaments, providing the stability associated with this structure [59,60,61,62], located on both sides of the bud neck and oriented along the mother–daughter axis [7,45,46] (Figure 2d). Interestingly, the LKB1-like kinase Elm1 associates with the septin hourglass throughout the cell cycle and plays a key role in its assembly and stability [63] (Figure 2d). This is achieved through the regulation of filament pairing via the septin-binding protein Bni5 [63], which associates with the C-terminal extensions of Cdc11 and Shs1 [64] (Figure 2b). The kinase Gin4 associates with Elm1, and they regulate each other initially during bud emergence and later during mitosis, ensuring that septin structures are assembled and remodeled in time to fulfill their specific functions at different stages of the cell cycle [65] (Figure 2d).

Prior to cytokinesis, the septin hourglass transforms into a “transitional hourglass”, where the paired filaments are interconnected perpendicularly by periodic single septin filaments arranged circumferentially on the side proximal to the membrane, forming a septin gauze [62]. At the onset of cytokinesis, the septin hourglass undergoes significant architectural reconfiguration, dividing into two distinct rings (Figure 2d). This separation allows the actomyosin ring to access the plasma membrane and initiate ingression [7,45,46]. Septin filament disassembly and reorganization drive the new circumferential arrangement of paired and single septin filaments to form a double ring, with filaments rotating 90° [59,60,62,66]. Two proteins, the Rho guanine-nucleotide-exchange factor (RhoGEF) Bud3 and the anillin-like protein Bud4, are essential for the transition of septin to double-ring structures but are not required for actomyosin ring contraction [66]. This septin ring organization is driven by the signaling cascade MEN [67]. Hof1, a component of the actomyosin ring as detailed below, is a critical target of MEN, and Hof1 phosphorylation induces septin ring splitting and Hof1 translocation from septins to the actomyosin ring [68,69,70,71].

## 3. Building a Contractile Ring

The actomyosin ring, responsible for the physical separation of the cytoplasm once shared by mother and daughter cells, is formed by numerous proteins whose assembly at the bud neck starts at the presumptive bud site [4,5]. The core of the actomyosin ring consists of actin filaments and the type II myosin, Myo1, which plays a scaffolding role in the assembly of the cytokinetic machinery [72]. Additionally, the recruitment and assembly of other cytokinetic factors occur stepwise throughout the cell cycle and are regulated by cell cycle signals [4,5].

**Figure 3:**
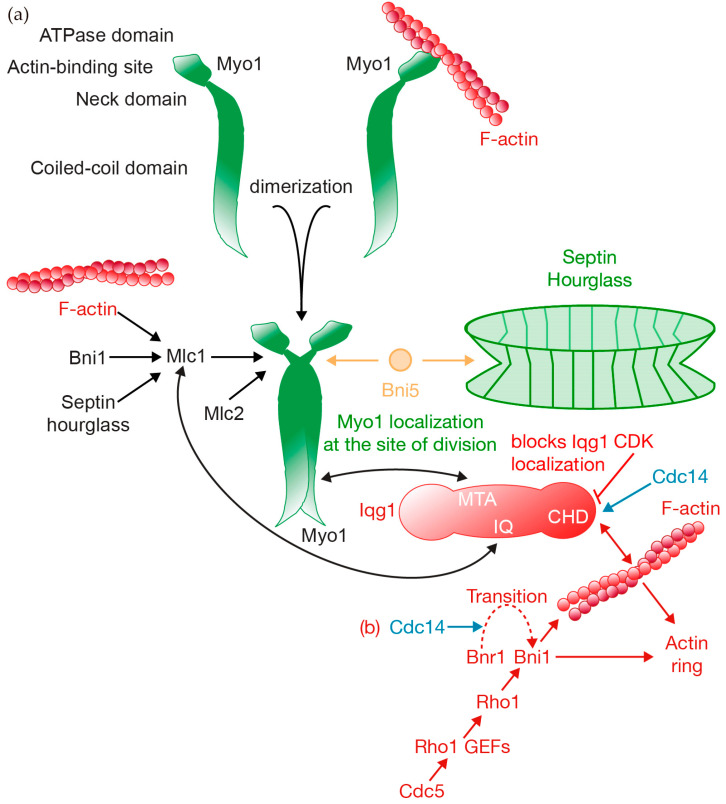
Myo1 plays a critical scaffolding role in assembling the cytokinetic machinery. (**a**) Myo1 is a motor protein, which contains distinct domains as depicted: an ATPase domain and an actin-binding site, a neck region that binds myosin light chains, and a coiled-coil tail domain responsible for myosin dimerization. A network of proteins regulates Myo1 localization and dynamics at the site of division. (**b**) One such protein is Iqg1, whose localization is interdependent with Myo1. Together, Iqg1 and formins drive actin ring assembly, a process inhibited by CDK kinase activity, which must be downregulated to complete the final assembly of the actomyosin ring.

Myo1 is a motor protein that consists of several domains: the globular head, which contains the ATPase domain and an actin-binding site; the neck domain, where myosin light chains bind; and a coiled-coil tail domain, which is involved in the dimerization of myosin [73,74] (Figure 3a). In budding yeast, the Myo1 tail alone is sufficient to support cytokinesis, a feature not observed in fission yeast [75]. This suggests the significance of the actomyosin ring not only for its contractile force but also in coordinating other processes, as discussed below. Myo1 forms a complex with an essential myosin light chain, Mlc1, and a regulatory light chain, Mlc2 [76] (Figure 3a). Myo1 appears to form filaments during cytokinesis [62,66]. Myo1 localizes to the bud site shortly before bud emergence and remains there after mitotic spindle disassembly [36,77] (Figure 1a). Moreover, the localization of Mlc1 to the site of division depends on the septin hourglass and is mediated by Myo1 [76,78] (Figure 3a). The localization of Mlc1 at the site of division during cytokinesis depends on filamentous actin (F-actin) and the formin Bni1 [78] (Figure 3a). However, the localization of Mlc2 to the site of division depends exclusively on Myo1 [76] (Figure 3a).

A specific region of Myo1 interacts with the septin-binding protein Bni5, which recruits Myo1 to the bud neck early in the G1 phase (Figure 3a). This interaction is crucial for maintaining Myo1 at the bud neck until the onset of cytokinesis [73]. Consequently, Bni5 serves as the immediate molecular connection facilitating the linkage of Myo1 to the septins (Figure 3a). The localization of Myo1 at the division site from the beginning of anaphase through to the completion of cytokinesis is dependent on the IQGAP protein, Iqg1 [73] (Figure 3). Consequently, both Bni5 and Iqg1 contribute to the localization of Myo1 during anaphase (Figure 3). Bni5 degradation and Iqg1 cell cycle-regulated expression determine the change between the two proteins [79,80,81].

Fluorescence recovery after photo-bleaching (FRAP) experiments has elucidated the dynamic nature of Myo1 at the division site, which becomes immobilized shortly before the onset of cytokinesis [72]. This observation suggests that Myo1 assumes a critical scaffolding role in the formation of the cytokinetic machinery. Following Myo1 dynamics by quantitative time-lapse imaging, Okada and colleagues have shown that the onset of actomyosin ring contraction coincides with the transition of the septin hourglass into a double ring [77]. Interestingly, super-resolution three-dimensional structured illumination microscopy (3D-SIM) has determined that both the actomyosin ring and septins are physically separated at the onset of cytokinesis [67], facilitating actomyosin ring contraction. The rate of constriction was initially slow, transitioning to a faster mode after the appearance of the septin double ring [77].

Given that the localization of Myo1 during cytokinesis depends on another component of the actomyosin ring, Iqg1 [73], it is highly plausible that Iqg1 functions both as a scaffold and in anchoring the myosin ring to the plasma membrane. Iqg1 is recruited to the division site during late anaphase via its IQ repeats, which are capable of interacting with Mlc1 [82,83,84] (Figure 3b). Additionally, Iqg1 uses a myosin-II tail-associated (MTA) domain to bind to Myo1 (Figure 3b), localizing Iqg1 to the site of division and regulating actomyosin ring assembly and function [85]. Iqg1 accumulates at the site of division during G2/M, peaking during actomyosin ring contraction and disappearing thereafter [77]. Cdk1-dependent phosphorylation of Iqg1 blocks its localization at the site of division (Figure 3b). A non-phosphorylatable version of Iqg1 localizes prematurely, together with actin and other components of the actomyosin ring like the Hof1 protein [86], which indicates the crucial role of Cdk1 in the regulation and timing of cytokinesis.

Iqg1 contains an amino-terminal calponin homology domain (CHD), which binds actin filaments. This interaction is essential for the assembly of the actin ring and is believed to facilitate the crosslinking of actin filaments [73,79,80,82,85,87]. The CHD plays a role in Iqg1 localization at the site of division as the lack of CHD alters that localization [85]. Both domains in Iqg1, CHD and MTA, collaborate to increase localization and specificity at the division site through their associations with Myo1 and actin filaments [85]. Additionally, both CHD and MTA regulate the assembly, constriction, and disassembly of the actomyosin ring [85].

Two other proteins, the formins Bni1 and Bnr1, are known to nucleate actin filaments, forming both actin cables and the actin ring [68,88,89,90,91] (Figure 3b). Iqg1 and formins operate both in parallel and independently in the assembly of the actin ring [90] (Figure 3b). Bnr1 is recruited to the site of division in a septin-dependent manner, extending from the G1 phase to telophase, while Bni1 localizes from telophase until the completion of cytokinesis [92,93]. This pattern of localization is consistent with the predominant role of Bni1 in the formation of the actin ring [68]. The transition from Bnr1 to Bni1 is controlled by the phosphatase Cdc14 [94] (Figure 3b). The function of formins is regulated by the small GTPase Rho1, which is recruited to the division site by its guanine nucleotide exchange factors (GEFs) that are phosphorylated by Cdc5 (Polo-like kinase) [95] (Figure 3b). GTP-bound Rho1 interacts with and activates the formins, thereby promoting the assembly of the actin ring, a process in which Rho1 is essential [88,89,90,95,96,97]. Furthermore, tropomyosins and profilin control the assembly and stability of the actin cables and actin ring; a lack of tropomyosin function promotes defects in actin ring assembly [89,90,98,99]. Rho1 mediates the recruitment to the site of division of one of the tropomyosins [95].

## 4. Ingression Progression Complexes (IPCs)

**Figure 4:**
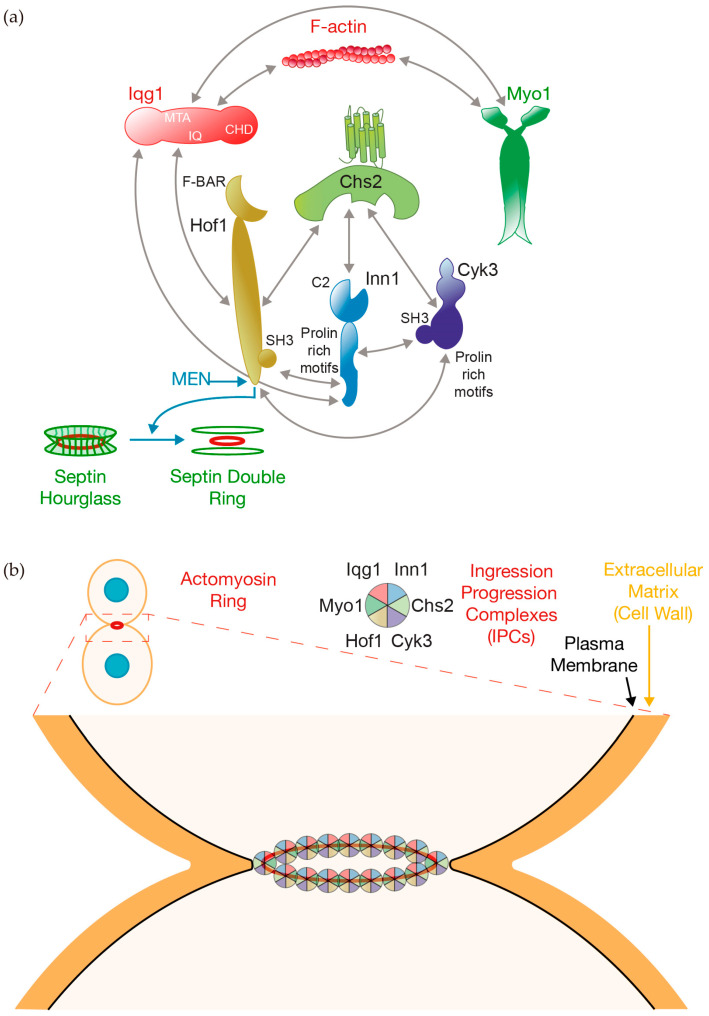
Members of the Ingression Progression Complexes (IPCs) in budding yeast cells. (**a**) The six proteins included in the IPCs are Myo1, Iqg1, Hof1, Inn1, Cyk3, and Chs2. This illustration depicts the interactions between them and highlights the key domains involved in these interactions. Additionally, it represents the role of the Mitotic Exit Network (MEN) in promoting the transition of the septin hourglass to a double ring via Hof1 phosphorylation. (**b**) IPCs localize at the site of division to coordinate actomyosin ring contraction, plasma membrane ingression, and extracellular matrix remodeling.

Myo1 is a key member of the so-called Ingression Progression Complexes (IPCs) that coordinate actomyosin ring contraction, plasma membrane ingression, and extracellular matrix remodeling [8] (Figure 4). The other components of the IPCs are the previously described Iqg1, along with the F-BAR protein Hof1, the transglutaminase-like domain-containing protein Cyk3, the C2 domain-containing protein Inn1, and the chitin synthase II Chs2 [8] (Figure 4). Together, they form a network of interactions. As described above, Myo1 binds to Iqg1 (Figure 3), which contains IQ repeats that interact with Hof1 [84] (Figure 4a). Additionally, Myo1 directly binds with Hof1, which localizes to the site of division in a complex manner [69,100] (Figure 4a).

Hof1 is characterized by the presence of an F-BAR domain within its N-terminal region and an SH3 domain at its C-terminus (Figure 4a), each of which has been demonstrated to be important for the dynamics and function of the Hof1 protein [69,100]. Hof1 localization peaks at three distinct moments during cytokinesis [77]. The first peak coincides with the transition of the septin hourglass to a double ring [77], followed by a second peak that corresponds with the onset of actomyosin ring contraction. At this stage, Hof1 shifts from the septin hourglass to the actomyosin ring [69], and the rest of the IPC components are recruited [77]. As described above, MEN-induced Hof1 phosphorylation drives septin ring splitting (Figure 4a), and the localization change of Hof1 [68,69,70,71]. This phase has been described as the fast phase of actomyosin ring constriction, in contrast to the last peak of Hof1 at the cleavage site, which appears to control the timely formation of the secondary septum [77,101]. Furthermore, Hof1 shares a role with Rvs167 in actin ring assembly and Iqg1 recruitment to the bud neck [102].

Following the stepwise IPC assembly, the SH3 domain of Hof1 is able to interact with proline-rich motifs situated at the C-terminus of Inn1 [102,103,104] (Figure 4a). Inn1 contains four such proline-rich motifs (PXXP) (Figure 4a). Together with Hof1, Inn1 also binds to Iqg1 [103] and Cyk3, the latter through the SH3 domain of Cyk3 at its N-terminus [24,102,104,105] (Figure 4a). Additionally, the SH3 domain of Hof1 binds to a proline-rich segment within Cyk3 [106] (Figure 4a). Hof1 directly interacts with Chs2 and stabilizes the chitin synthase at the cleavage site [100] (Figure 4a). Inn1 and Cyk3 directly bind to the catalytic domain of Chs2 [8] (Figure 4a). All six IPC components—Myo1, Iqg1, Hof1, Inn1, Cyk3, and Chs2—interact during cytokinesis to form larger complexes that coordinate the late steps of cell division in budding yeast [8] (Figure 4b). Inn1, Cyk3, and Chs2 are the last IPC members to localize to the site of division, coinciding with the onset of the fast-phase actomyosin ring constriction [77].

## 5. Actomyosin Ring Constriction

Following GFP-tagged Myo1 using time-lapse microscopy, it has been determined that the constriction rate starts slowly at 0.02 ± 0.02 μm/min for approximately 4 min, then increases significantly to approximately 0.18 ± 0.05 μm/min for about 6 min [77]. This second and faster phase accounts for around 90% of the ring constriction and occurs once the transition of the septin hourglass to a double ring is completed, suggesting its importance in enabling actomyosin ring constriction [66,67].

In budding yeast, the process of ring constriction is linked to the inward growth of the septum [4,5] (Figure 4b). The *MYO1* gene is essential in some strains, although in other genetic backgrounds, cells can perform cell division by producing a remedial septum [10,36,107,108,109]. Interestingly, the myosin heads responsible for interacting with actin filaments are not essential and are not required for ring constriction or cytokinesis, unlike what occurs in other types of cells like fission yeast [73,75]. Consequently, septum growth and abscission can still occur in the absence of forces exerted by Myo1 in budding yeast. It is hypothesized that the indispensable tails of Myo1 may function as scaffolds in this process. Furthermore, using a quantitative microscopic model, it was proposed that actomyosin ring constriction is based on filament sliding driven by actin depolymerization and myosin II motor activity [110]. A mathematical model, supported by experimental measurements, suggests that actin depolymerization is the prevailing mechanism for ring constriction. The model also predicts a constant contraction rate regardless of the initial ring size [110].

The ubiquitin ligase known as the anaphase-promoting complex (APC) targets cell cycle regulators for degradation, inducing mitosis progression. Additionally, APC plays a role in the actomyosin ring disassembly during cytokinesis. When APC is defective, disassembly occurs at a normal rate, but aggregates of actomyosin ring proteins remain at the division site. Iqg1 is one of the APC targets [111].

## 6. Mechanism and Regulation of Primary Septum Deposition

**Figure 5:**
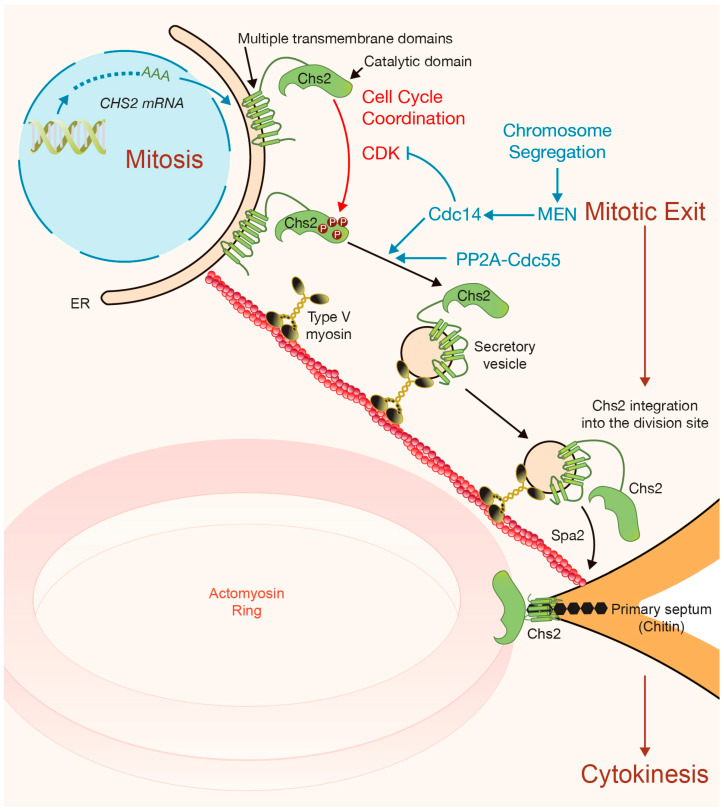
The dynamics and function of Chs2. Chitin synthase II (Chs2) is expressed during mitosis but initially retained in the endoplasmic reticulum (ER) due to CDK-dependent phosphorylation on its catalytic domain. Chs2 contains multiple transmembrane domains that anchor it to the plasma membrane. Upon chromosome segregation, the Mitotic Exit Network (MEN) becomes activated, triggering the release of the phosphatase Cdc14, which dephosphorylates Chs2. Additionally, the phosphatase PP2A-Cdc55 contributes to Chs2’s dephosphorylation. This promotes Chs2 incorporation into secretory vesicles, which are transported to the division site by type V myosin. At the division site, Chs2 integrates into the cytokinesis machinery, remaining connected to the plasma membrane—an essential feature for its function. Spa2 facilitates Chs2 incorporation. Once active, Chs2 synthesizes the primary septum, composed of chitin, extruding the chitin in a centripetal manner just behind the contracting actomyosin ring.

In budding yeast, Chs2 catalyzes the formation of a special cell wall structure known as the primary septum, which is made of chitin (Figure 5). Chs2 contains a catalytic domain flanked by a C-terminal region containing multiple transmembrane (TM) domains (Figure 5). Chs2 extrudes the chitin to the outside of the cell in a centripetal fashion just behind the contracting actomyosin ring [10,112,113] (Figure 5). Actomyosin ring constriction and ingression of the plasma membrane at the cleavage site are tightly coupled to primary septum formation [114] (Figure 5). Disruptions in any of these processes can affect the others [36,103,107,115,116]. Therefore, controlling the activity of chitin synthase Chs2 is crucial for successful cytokinesis. Regulatory mechanisms ensure the coordination of primary septum formation with cell cycle progression, initiating only after chromosome segregation and actomyosin ring assembly [114] (Figure 5).

The expression, recruitment, and enzymatic activity of chitin synthase Chs2 are tightly regulated in terms of timing and location [77,116,117,118,119,120,121] (Figure 5). Chs2 is synthesized during mitosis and retained in the endoplasmic reticulum (ER) until late anaphase, when it is transported to the site of division in secretory vesicles [116,119,120,121] (Figure 5). Toward the end of the cell cycle, mitotic forms of CDK must be downregulated to enable cell exit from mitosis, execute cytokinesis, and initiate a new cell cycle (Figure 5). Chs2 localization and function are highly cell cycle-regulated. Chs2 undergoes phosphorylation by Cdk1 in vivo [121] (Figure 5). During mitosis, when CDK activity is still high, Chs2 is expressed but retained at the ER due to phosphorylation by Cdk1 at four consensus Cdk1 sites at its N-terminus [119,120] (Figure 5). Dephosphorylation of Chs2 by Cdc14 drives Chs2 export from the ER to the site of division through the secretory pathway, facilitated by actin cables and type V myosin [116,117,121] (Figure 5). Furthermore, the PP2A-Cdc55 phosphatase controls mitosis and counteracts phosphorylation by the kinases Cdk1 and Cdc5 (Figure 5). PP2A-Cdc55 dephosphorylates Chs2, promoting correct actomyosin ring contraction and primary septum formation [122].

In addition to its role in promoting exit from mitosis, MEN controls correct cytokinesis by regulating components of the IPCs [26,27]. A lack of MEN function prevents efficient localization of Chs2 at the site of division, reducing chitin deposition [26]. The MEN kinase Dbf2 directly phosphorylates Chs2 on a single residue, which has been shown to be important for primary septum formation [27].

Chs2 localizes to the site of division a few minutes before spindle breakage and the start of actomyosin ring contraction [77]. Fluorescence Recovery After Photobleaching (FRAP) analysis showed that the initial localization of Chs2 is quite dynamic, with its delivery being completed within a few minutes of its arrival [72]. Subsequently, during cytokinesis, Chs2 becomes immobile, and this immobility is dependent on Myo1 [72].

The localization of Chs2 at the cleavage furrow appears to be insufficient for Chs2 to synthesize the primary septum [104]. Using cell membrane containing Chs2, chitin activity associated with Chs2 increased after trypsin treatment [112,123], suggesting that Chs2 requires activation in vivo once the protein is localized at the site of division. Over the last twenty years, we have gained further molecular details on how Chs2 is regulated and how important this activation is to ensure the precise coordination of actomyosin ring contraction, plasma membrane ingression, and primary septum formation.

**Figure 6:**
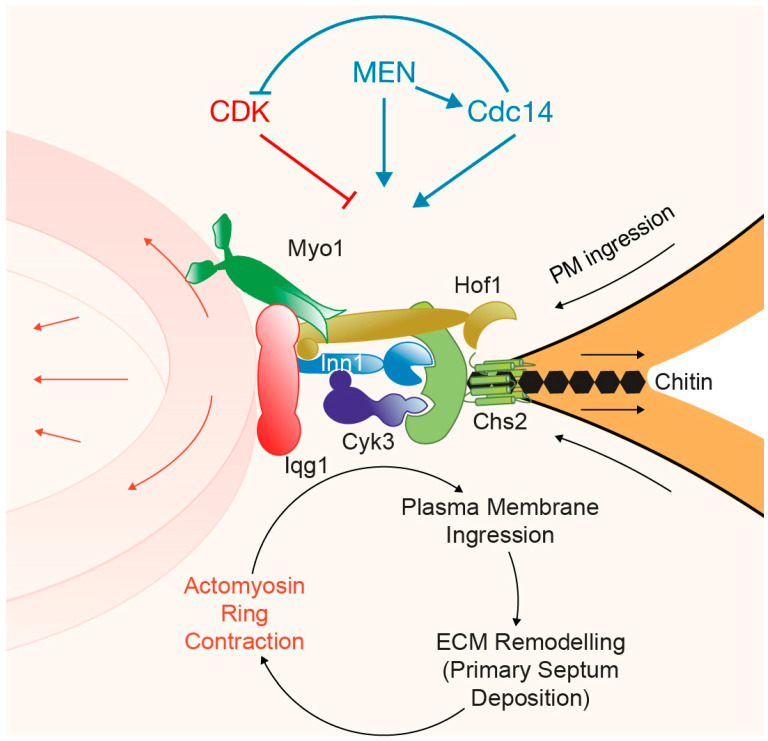
Coordination of actomyosin ring contraction, plasma membrane ingression, and extracellular matrix remodeling. The ingression progression complexes (IPCs) coordinate actomyosin ring contraction, plasma membrane ingression, and extracellular matrix remodeling. Illustration of the IPCs depicting their coordinated function to achieve successful cytokinesis. The three processes are interconnected, and the failure of one of them affects the others. The coordinated action of kinase activity associated with CDK (depicted in red) and MEN (depicted in blue), together with the phosphatase Cdc14 (depicted in blue), ensures cytokinesis occurs in a timely manner.

Three proteins directly regulate the activity of Chs2 at the site of division: Hof1, Inn1, and Cyk3 [8,103,104,124,125] (Figure 6). As described previously, Hof1 dynamics at the site of division are complex [69,100], and its initial localization occurs before Inn1, Cyk3, and Chs2 arrive at the cleavage site [77]. The other two components of the IPCs, Myo1 and Iqg1, are also recruited to the site of division prior to Inn1, Cyk3, and Chs2 [77]. Interestingly, the cell polarity protein Spa2 is recruited to the site of division, where it collaborates with the secretory vesicle system (Figure 5) and proteins Hof1 and Cyk3 to promote the incorporation of Chs2 into the IPCs [9]. The recruitment of Inn1, Cyk3, and Chs2 coincides with fast-phase actomyosin ring constriction [77], which indicates that the final formation of IPCs drives this fast mode of constriction. It is the coordinated action of all IPC components that promotes the coordination of actomyosin ring contraction, plasma membrane ingression, and extracellular matrix remodeling [8,104,105,126] (Figure 6). The interaction of Inn1 with Cyk3 is inhibited by Cdk1, and therefore the phosphatase Cdc14 dephosphorylates Inn1, allowing Inn1 to bind to Cyk3 and drive cytokinesis [24] (Figure 6). Similarly, Cdc14 dephosphorylates Cdk1 sites around the Iqg1 CHD to promote actin ring formation earlier in cytokinesis [127] (Figure 3).

Cyk3 regulates the function of Chs2. An elevated dosage of Cyk3p increases Chs2-dependent chitin synthesis and promotes the formation of primary-septum-like structures at the bud neck, membrane ingression, and secondary septum formation during cytokinesis [26,27,104]. A proline-rich motif in Cyk3 mediates direct interaction with the SH3 domain of Hof1 (Figure 4), which contributes to the formation of primary septum and plasma membrane ingression [126] (Figure 6).

Another component of the IPCs, the protein Inn1, directly regulates the function of Chs2, making it essential for coordinating actomyosin ring contraction, plasma membrane ingression, and primary septum deposition [103,104]. Inn1 contains a C2 domain at its amino terminus (Figure 4a), which is crucial for plasma membrane ingression, while the remaining protein is necessary for the timely localization of Inn1 at the bud neck [103]. Dominant suppressor mutations in Chs2 suppress the defects in plasma membrane ingression caused by an inactive form of the C2 domain of Inn1. These mutations are situated at the catalytic domain and display increased chitin synthase activity [125]. Indeed, the C2 domain of Inn1 and the transglutaminase-like domain of Cyk3 enhance the chitin synthase activity associated with Chs2 [8]. Cyk3 plays a crucial role in releasing the block on Chs2 activity imposed by the C-terminus of Inn1, preventing premature activation of Chs2 at the division site [8]. Interestingly, Cyk3 plays a coordinating role by inhibiting late steps during cytokinesis—secondary septum formation—while promoting earlier steps during cell division [128]. MEN targets Cyk3, together with Inn1 and Chs2, to the site of division [26].

## 7. Concluding Remarks

Cytokinesis is an essential process for the successful completion of cell division, ensuring the proper segregation of genetic material and cellular components into daughter cells. Comparative studies in model organisms and higher eukaryotes have significantly advanced our understanding of the general principles of cytokinesis. Table 1 highlights the orthologs involved in cytokinesis in budding yeast, fission yeast, and pathogenic *Candida albicans*. In *S. cerevisiae*, the intricate coordination of the actomyosin ring, plasma membrane ingression, and extracellular matrix remodeling, as detailed above, provides a model to understand the fundamental mechanisms of cytokinesis in human cells (Figure 6).

Several components of the Ingression Progression Complexes are conserved across evolution. Budding yeast has a single isoform of myosin type II, while *Schizosaccharomyces pombe* exhibits two (Table 1), and mammalian cells display increased complexity with three non-muscle myosin-II heavy chains (*MYH9*, *MYH10*, and *MYH14*) [74]. While the in vivo assembly, function, and regulation of the yeast myosin II are well understood, knowledge about its mammalian counterparts is still limited. Similarly, Iqg1 is conserved, with one ortholog in fission yeast (Table 1) and three in higher eukaryotes (*IQGAP1*, *IQGAP2*, and *IQGAP3*) [87,129]. Depletion of IQGAP proteins results in defects in cytokinesis, indicating their crucial role during cell division [130]. As with myosin II, further research is needed to fully elucidate their specific roles. Another conserved IPC component is Hof1, for which the protein PSTPIP1 shares domain structure [131,132]. Alterations in PSTPIP1 regulation lead to defects in cytokinesis [132,133], suggesting its involvement in cell division.

Conversely, *S. pombe* possesses a clear ortholog of Inn1, known as Fic1 [134] (Table 1). While no direct human ortholog for Inn1 has been identified, certain human proteins exhibit similarities, such as a C2 domain and an unstructured C-terminal region. Future studies could reveal whether Inn1’s role in cytokinesis is preserved in human cells or if alternative mechanisms have evolved to coordinate the final stages of cell division. Furthermore, while Cyk3 has been identified in fission yeast [135] (Table 1), no animal ortholog exists. Similarly, Chs2, which is essential for the synthesis of the primary septum chitin in budding yeast, has no mammalian counterpart due to the lack of chitin in higher eukaryotes. Loss of Chs2 function results in cytokinesis defects [116]. Interestingly, the glycosyltransferase Bgs1 in fission yeast fulfills a role similar to that of Chs2 in budding yeast, despite producing a different polysaccharide. Defects in Bgs1 lead to cytokinesis failures [136,137]. Remarkably, similar defects are observed in *Caenorhabditis elegans* and mouse models when glycosyltransferases are depleted during early embryogenesis, emphasizing the conserved role of ECM remodeling in cytokinesis [138,139]. These findings suggest that although specific IPC proteins may differ, the cellular strategies underlying successful cell division are likely more conserved than currently appreciated.

Future research should focus on further elucidating the molecular details of the mechanics of ring contraction, including understanding how actin and myosin interactions are fine-tuned during the contraction process. Additionally, it is important to gain deeper insights into how the actomyosin ring anchors to the plasma membrane and to shed light on the coordinated and specific incorporation of secretory vesicles at the moment of contraction. Identification of all members of the IPCs would help to understand if the way in which budding yeast coordinates actomyosin ring contraction, plasma membrane ingression, and extracellular matrix deposition is fully conserved. Studying these processes in yeast provides valuable insights into the fundamental principles of cell division and their relevance to human health. This is particularly significant in understanding diseases such as cancer, where defects in cytokinesis can result in genomic instability and uncontrolled cell proliferation.

The study of yeast cytokinesis holds significant translational potential for human health. As previously mentioned, the mechanisms of cytokinesis are conserved across eukaryotes, from yeast to mammals. However, it is likely that certain specific enzymatic activities, such as those of glycosyltransferases like Chs2, may be shared predominantly among fungal pathogens like *Candida albicans*. As highlighted in Table 1, most of the proteins involved in cytokinesis, and described in this review, are conserved across these species. Therefore, understanding how model organisms like *S. cerevisiae* undergo cell division is crucial for developing targeted therapeutic strategies against fungal pathogens.

**Table 1:** Genes discussed in this review and involved in the regulation of cytokinesis in *S. cerevisiae*, *S. pombe*, and *Candida albicans*. Additionally, the table includes references to *C. albicans* virulence studies tested in mouse models.

*S. cerevisiae*	Type of Protein/Function	*S. pombe*	*C. albicans*	*Candida albicans* Virulence Studies
*BNI1*	Formin	*cdc12*	*BNI1*	mouse intravenous infection [140]
*BNI5*	Bud protein	nda	*BNI5*	nda
*BUD3*	Bud protein	nda	*BUD3*	nda
*BUD4*	Bud protein	nda	*INT1*	nda
*CDC28*	CDK1	*cdc2*	*CDC28*	nda
*CDC3*	Septin	*spn1*	*CDC3*	nda
*CDC5*	Polo kinase	*plo1*	*CDC5*	
*CDC10*	Septin	*spn2*	*CDC10*	mouse intravenous infection [141]
*CDC11*	Septin	*spn3*	*CDC11*	mouse intravenous infection [142]
*CDC12*	Septin	*spn4* *spn6*	*CDC12*	nda
*CDC14*	MEN pathway	*clp1*	*CDC14*	nda
*CDC15*	MEN pathway	*cdc7*	*CDC15*	nda
*CDC42*	GTPase	*cdc42*	*CDC42*	nda
*CDC55*	Phosphatase PP2A	*pab1*	*CDC55*	nda
*CHS2*	Chitin synthase	*chs1* *chs2*	*CHS1*	mouse intravenous infection [143,144]
*CYK3*	Contractile ring Assembly	*cyk3*	*CYK3*	nda
*DBF2*	MEN pathway	*sid2*	*DBF2*	nda
*DBF20*	MEN pathway	*sid2*	nda	nda
*ELM1*	Morphogenesis	*ssp1*	*ELM1*	nda
*GIC1*	GTPase binding	nda	nda	nda
*GIC2*	GTPase binding	nda	nda	nda
*GIN4*	Septin ring assembly	*cdr2* *cdr1*	*GIN4*	nda
*HOF1*	Contractile ring assembly	*cdc15* *imp2*	*HOF1*	nda
*INN1*	Contractile ring assembly	*fic1*	*INN1*	nda
*IQG1*	IQGAP	*rng2*	*IQG1*	nda
*MLC1*	Myosin essential light chain	*cdc4*	*MLC1*	nda
*MLC2*	Myosin regulatory light chain	*rlc1*	*C5_03370C_A*	nda
*MYO1*	Myosin II	*myo2* *myp2*	*MYO1*	nda
*RVS167*	Actin binding	*hob1*	*RVS167*	mouse oropharyngeal infection [145], mouse intravenous infection [146]
*SHS1*	Septin	*spn3*	*SEP7*	nda
*SPA2*	Polarisome component	*spa2*	*SPA2*	mouse intravenous infection [147]
*TEM1*	MEN pathway	*spg1*	*TEM1*	mouse intravenous infection [144]

Data for the table were sourced from www.yeastgenome.org (*S. cerevisiae*), www.pombase.org (*S. pombe*), and www.candidagenome.org (*C. albicans*) accessed on 10 September 2024. nda: no data available.

## Data Availability

No new data were created or analyzed in this study.
